# Experience with using second life for medical education in a family and community medicine education unit

**DOI:** 10.1186/1472-6920-12-30

**Published:** 2012-05-15

**Authors:** Elena Melús-Palazón, Cruz Bartolomé-Moreno, Juan Carlos Palacín-Arbués, Antonio Lafuente-Lafuente, Inmaculada García García, Sara Guillen, Ana B Esteban, Silvia Clemente, Ángeles M Marco, Pilar M Gargallo, Carlos López, Rosa Magallón-Botaya

**Affiliations:** 1 Family and Community Medicine Education Unit, Aragonese Health Service, Zaragoza I Zone, Eugenio Lucas 31-33, 50018, Zaragoza, Spain; 2 Education Service of the Department of Planning, Government of Aragon, Zaragoza, Spain; 3 Primary Care Administration, Aragonese Health Service, Zaragoza I Zone, Zaragoza, Spain; 4 Department of Medicine, University of Zaragoza, Zaragoza, Spain

## Abstract

**Background:**

The application of new technologies to the education of health professionals is both a challenge and a necessity. Virtual worlds are increasingly being explored as a support for education. Aim: The aim of this work is to study the suitability of Second Life (SL) as an educational tool for primary healthcare professionals.

**Methods:**

Design: Qualitative study of accredited clinical sessions in SL included in a continuing professional development (CPD) programme for primary healthcare professionals. Location: Zaragoza I Zone Family and Community Medicine Education Unit (EU) and 9 health centres operated by the Aragonese Health Service, Aragon, Spain. Method: The EU held two training workshops in SL for 16 healthcare professionals from 9 health centres by means of two workshops, and requested them to facilitate clinical sessions in SL. Attendance was open to all personnel from the EU and the 9 health centres. After a trail period of clinical sessions held at 5 health centres between May and November 2010, the CPD-accredited clinical sessions were held at 9 health centres between February and April 2011. Participants: 76 healthcare professionals attended the CPD-accredited clinical sessions in SL. Main measurements: Questionnaire on completion of the clinical sessions.

**Results:**

Response rate: 42-100%. Questionnaire completed by each health centre on completion of the CPD-accredited clinical sessions: Access to SL: 2 centres were unable to gain access. Sound problems: 0% (0/9). Image problems: 0% (0/9). Voice/text chat: used in 100% (10/9); 0 incidents. Questionnaire completed by participants in the CPD-accredited clinical sessions: Preference for SL as a tool: 100% (76/76). Strengths of this method: 74% (56/76) considered it eliminated the need to travel; 68% (52/76) believed it made more effective use of educational resources; and 47% (36/76) considered it improved accessibility. Weaknesses: 91% (69/76) experienced technical problems, while; 9% (7/76) thought it was impersonal and with little interaction. 65.79% (50/76) believed it was better than other distance learning methods and 38.16% (29/76) believed it was better than face-to-face learning.

**Conclusions:**

SL is a tool that allows educational activities to be designed that involve a number of health centres in different geographical locations, consequently eliminating the need to travel and making more effective use of educational resources.

## Background

The application of new technologies to the education of healthcare professionals is both a challenge and a necessity. Virtual worlds are increasingly being explored as a support for medical education and to respond to the problems found in conventional learning methods, such as time constraints and travel requirements.

Second Life (SL) has been used by companies and private groups as part of their Web 2.0 communication strategies [1]. It is a three-dimensional environment [2], consisting of a simulated Earth divided into regions, with private regions in the form of islands. It was created by Linden Lab, based in San Francisco, USA, in 2003 [3]. Access to SL requires the download of a free software program. People enter and interact in SL by means of avatars. Physical requirements for SL are a computer with Internet access, a loudspeaker and a headset with a microphone.

There are a number of communication channels in the virtual world; besides the visual image, there is the possibility of voice chat, text chat and instant messaging.

The virtual world enables healthcare professionals in different locations to connect with each other without the physical limitations of the real world, which is an important consideration to take into account when designing educational activities. SL has been described as a useful model for simulation [4] and one that offers new ways of communicating health information [5]. The importance of professional cooperation between health professionals in SL has also been suggested [4,6]. Moreover, in addition to its design, it has other unique features that make it suitable for the education of healthcare professionals [7]. The virtual world offers opportunities for interaction between health workers and as a simulator of situations. The first International Virtual Association of Surgeons meeting was held in SL in 2008 and was attended by 47 delegates from five countries [8].

Interest has been growing in recent years among medical and health-related communities in the use of SL for dissemination, education and professional development [9]. In Spain, the Spanish Society for Family and Community Medicine (SEMFyC) owns **“*Isla de la Salud*”** (Health Island) in SL, which is used to host educational activities [10]. Likewise, the greater sensation of “presence” in an activity has a positive influence on learning compared to other virtual methods [11]. Consequently, we believed that it could be of significant use as a platform for educational activities in order to overcome the problem of distance between health centres in our zone, up to 134 km between the most remote.

The aim of this project was to explore the possibility of using SL for health education, through accredited clinical sessions – educational presentations given by primary care practitioners – as part of a continuing professional development (CPD) programme to teach junior doctors and other primary healthcare professionals in the same administrative zone, and to gain an understanding into its strengths and limitations.

## Methods

### Design

The Zaragoza I Zone Family and Community Medicine Education Unit (EU) chose to incorporate new technologies into its educational activities. SL was chosen as a means to overcome the particular obstacle posed by the dispersion of health centres in this zone by doing away with the need for healthcare professionals to travel long distances for such activities, particularly given the time constraints faced by many of them.

All primary care centres in our zone have Internet access in the computers located both in GP surgeries and the libraries/seminar rooms where clinical sessions are held. The SL program was installed on the computers in each library/seminar room of the participating health centres by the zone’s primary care administration and IT service.

SEMFyC authorized use of the virtual open-air auditorium on Health Island for this activity. A screen was designed in the auditorium to enable sessions to be presented in PowerPoint, and a podium was created for presenters. Access to the space was open to any interested person.

**Figure 1:**
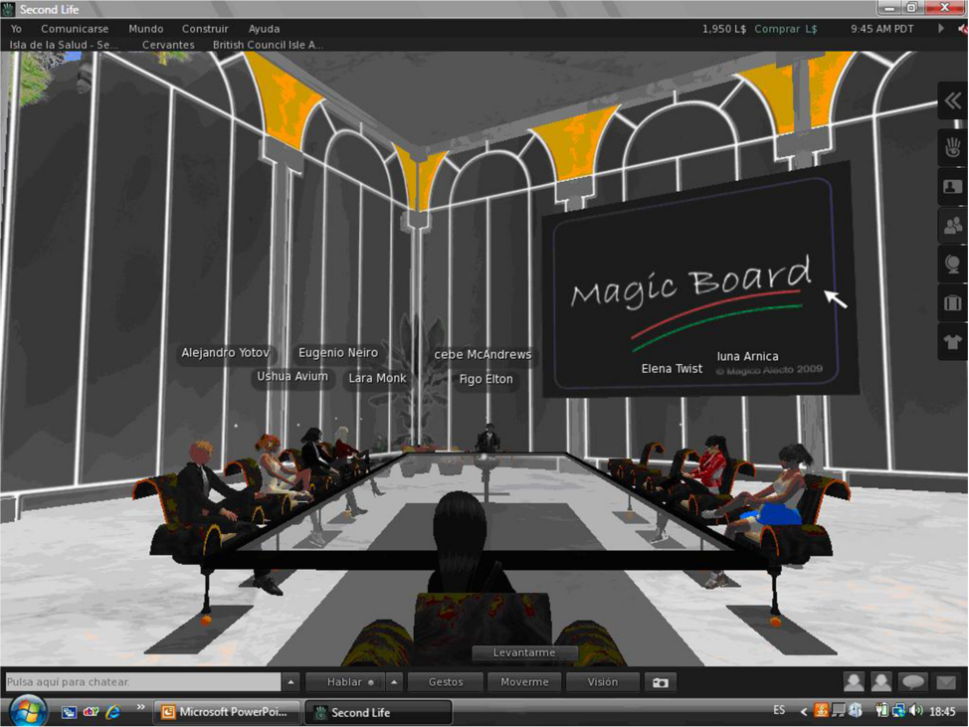
**Session in Second Life.** One of the training workshops held with teaching staff and students in Second Life.

As most of the healthcare professionals involved were unfamiliar with this tool, the initial commitment included training in the use of SL and the facilitating of clinical sessions on this platform, for which specific resources (handbooks [12] and explanatory videos [13]) were designed. Two CPD-accredited face-to-face workshops were held to train 16 healthcare professionals from 9 health centres (see Figure 1) as teachers in SL.

**Figure 2:**
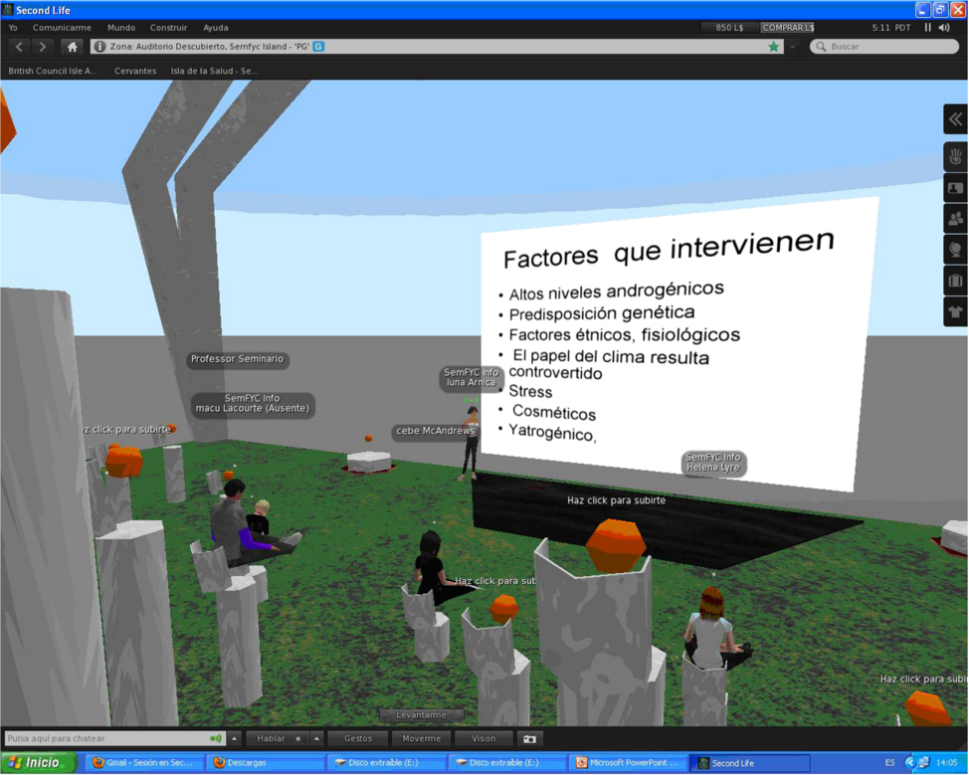
**Session in Second Life.** Meeting in Second Life of all students and teaching staff to set up the clinical sessions schedule.

On completion of the workshops, a schedule was designed for non-accredited trial sessions (see Figure 2), which were held between May and November 2010 on Fridays at 2 pm with a 30-minute duration. The clinical sessions were to cover subject matter in the fields of preventive medicine, family and community medicine, prescription drugs and new technologies. The sessions were structured around a 30-minute presentation by the teacher, followed by 5 minutes of questions and answers and 10 minutes of debate. The particular topics covered by the sessions were: updating knowledge on breast and cervical cancer screening; updating knowledge on vaccination and screening for infectious diseases in immigrant patients, among others (preventive); acute pulmonary oedema and results of the evaluation of COPD patient monitoring, among others (medical); updating the use of proton pump inhibitors and drug intervention to help patients stop smoking (prescription drugs); and use of the new Intranet portal and creation of an electronic portfolio, among others (new technologies). All the content presented in the sessions, together with additional material of interest (articles, clinical practice guidelines, etc.) were uploaded to Google Site to allow continued access once the session was finished. The presentations and additional materials are available (in Spanish) via this link: https://sites.google.com/site/sesionesclinicasudmfycs1z/calendario-actividades.

Each of the teachers who had received training in the workshops prepared a clinical session and attended all the sessions prepared by the others. They each presented a clinical session from their respective health centres. The sessions were held in the health centre library/seminar room in order to accommodate any other health centre personnel wishing to attend. Any participants away from their health centres at the time were able to connect via their laptops. The clinical sessions were held on a voluntary basis and were not remunerated.

Any incidents, advantages, strengths and weaknesses noted during a session were reported once it was over. This enabled experience to be gained in the operation of the new tool ( http://www.youtube.com/watch?v=JbDnWvTogkI, http://www.youtube.com/watch?v=FgR3DANrShc, http://www.youtube.com/watch?v=Ey5F01as2Aw).

Once this series of trial clinical sessions was completed, a full schedule of 9 open-access, CPD-accredited clinical sessions was designed to run between February and April 2011. The subject areas and structure of the sessions were maintained. This official programme specifically covered these topics: prescribing exercise for the prevention of cardiovascular disease (preventive); osteoporosis and treating patients with acute asthma (medical); prescription by active ingredient and updating knowledge on treatments for degenerative osteoarthritis (prescription drugs); and new technology platforms for medical teaching and new research tools (new technologies). As with the trial sessions, all the content presented in this series of sessions, together with additional material of interest were uploaded to Google Site to allow continued access once the session was finished. These presentations and additional materials are available (in Spanish) via this link: https://sites.google.com/site/sesionesclinicasudmfycs1z/sesiones-clinicas-2011-2012 A survey was designed for completion by participants after the last session was held.

Attendance was recorded with a photograph of the avatars, complemented by an attendance sheet that was signed before and after each session at each of the participating health centres.

Participating Aragonese Health Service operated health centres: Urban: Arrabal, Actur Oeste, Actur Norte, Actur Sur, La Jota, Parque Goya (Zaragoza 1 Zone) and Huesca (Huesca Zone). Rural: Luna (Zaragoza 1 Zone) and Híjar (Teruel Zone).

### Ethical aspects

Informed consent: Verbal information was provided. Subjects had sufficient opportunity to inquire about the details of the study. The rules governing confidentiality contained in the Helsinki Declaration were followed. Only researchers had access to the responses from participants. These responses were anonymous and no personal information was recorded in the database. Each response was given a code as the only form of identification appearing in both the database and the information connected with the study in order to guarantee confidentiality. The project was not presented to the Clinical Research Ethics Committee of Aragon for approval for the following reasons: it was not a clinical study; it was not an observational prospective observational post-authorization safety study involving drugs; it was not a research project involving interventions in human beings; no human biological or embryonic samples were used; there was no genetic analysis involved; there was no collaboration with biobanks; and no personal data were used.

### Inclusion criterion

Healthcare professionals from primary care health centres in the Aragonese Health Service who received training for presenting clinical sessions in SL.

### Participant selection procedure

Where there was at least one health professional present with training in the use of SL for clinical sessions, all personnel from the participating health centre were offered the opportunity to enrol in the CPD-accredited clinical sessions. A total of 76 healthcare professionals enrolled.

### Interventions performed

CPD-accredited clinical sessions in SL, after previous training workshops and non-accredited trial clinical sessions.

### Measurement techniques

One questionnaire completed by a member of the EU after the sessions: Could everybody access Second Life?; Were there any problems with sound or image?; Was the voice or text chat used to participate or express doubts?; Were hyperlinks and material referred to during the session sent?; and Were there any incidents? Another questionnaire was completed by clinical session participants: Do you consider this to be an interactive method?; How did you feel about interacting with other health centres?; What did you think about the interaction with teaching staff?; What did you think about the interaction with other healthcare professionals? How did you feel about not having to travel?; Do you think it facilitates the exchange of medical information?; Do you think it facilitates learning?; Do you think it facilitates debate?; Did you feel comfortable attending the sessions?; Did you feel comfortable asking questions?; Did you feel more comfortable than in face-to-face sessions?; What is your opinion of the text chat?; What is your opinion of the voice chat?; Does it matter to you to be represented by an avatar?; Did you think it was positive being able to see the speaker, setting and presentations?; In general, did you like this tool?; Do you consider it better than other distance learning methods?; Do you consider it better than face-to-face learning methods?; What are strengths of this tool?; and What are the weaknesses of this tool?

### Statistical analysis

Descriptive study of the results.

## Results

The results related to the technical support and possible errors in SL taken from a questionnaire for completion by each health centre after each clinical session are shown in Table 1.

**Table 1:** Questionnaire on the technical problems encountered by health centres after the clinical sessions

**Technical areas analysed**	**% (n/N)**	**Comments**
Access to SL	2 health centres (2/9) were unable to access 2 sessions	
Sound problems	0% sessions (0/9)	
Image problems	0% sessions (0/9)	
Voice chat	100% sessions (9/9)	After the session
Text chat	100% sessions (9/9)	During the session
Voice chat doubts	100% sessions (9/9)	After the session
Text chat doubts	100% sessions (9/9)	During the session
Sending of additional information	1 web page link	
	Brand names for active ingredients on three occasions	
	1 protocol	
Incidents	Intruder avatars appeared during several sessions without causing incidents	2 healthcare professionals and 1 non-healthcare professional

SL: Second Life.

The opinions of the 76 health professionals that participated in the CPD-accredited clinical sessions were submitted via text chat. Their opinions on the use of SL in teaching are given in Tables 2 and 3. Their responses regarding what the virtual environment offers and comparisons with other face-to-face and distance learning methods are given in Table 4. Finally, their responses regarding the strengths and weaknesses of SL are shown in Table 5.

**Table 2:** Opinion questionnaire after clinical sessions: Usefulness of Second Life for teaching I

**Question**	**Answer**	**% (n/N)**
Do you consider this to be an interactive method?	“yes”	68.42% (52/76)
	“partly”, “perhaps”	31.58% (24/76)
How did you feel about interacting with other health centres?	“good”, “very good”, “excellent”,“perfect”, “more participation”, “it's very important and it's also in real time”	100% (76/76)
What did you think about the interaction with teaching staff?	“good”, “adequate”	85.53% (65/76)
	“little”, “minimal”	14.47% (11/76)
What did you think about the interaction with other health professionals?	“adequate”, “good”, “it works very well”	43.42% (33/76)
	“almost non-existent”, “we wouldn’t have participated even if we could have”	56.58% (43/76)
Do you think it facilitates the exchange of medical information?	“yes”	77.63% (59/76)
	“several people with a single connection limited participation”	22.37% (17/76)
	no response	7.89% (6/76)
Do you think it facilitates learning?	“yes”, “of course” and “it’s a comfortable way”	100% (76/76)
Do you think it facilitates debate?	“yes”, “more when participation was on an individual basis”	22.37% (17/76)
	“somewhat”, “it makes debate a little difficult”, “not so much, but it could”	60.53% (46/76)
	“no”	9.21% (7/76)
	no response	7.89% (6/76)

SL: Second Life.

**Table 3:** Opinion questionnaire after clinical sessions: Usefulness of Second Life for teaching II

**Question**	**Answer**	**% (n/N)**
How did you feel about not having to travel?	“very good”, “great”, “fantastic”, “wonderful”	92.11% (70/76)
	no response	7.89% (6/76)
Did you feel comfortable attending the sessions	“yes”, “very comfortable”, “I was able to attend from work and home”	92.11% (70/76)
	no response	7.89% (6/76)
Did you feel comfortable asking questions?	“yes”, “it’s less embarrassing than in public”	92.11% (70/76)
	no response	7.89% (6/76)
Did you feel more comfortable than in face-to-face sessions?	“yes”	30.26% (23/76)
	“there’s no problem either this way or face-to-face”	5.26% (4/76)
	“no”	55.26% (42/76)
	no response	9.21% (7/76)
What is your opinion of the text chat?	“it’s good”, “very useful”	86.84% (66/76)
	“it takes a bit of getting used to the system”	9.21% (7/76)
	no response	3.95% (3/76)
What is your opinion of the voice chat?	“very good”, “very useful”	43.42% (33/76)
	“we weren’t able to do it”, “it isn’t enough for that”	14.47% (11/76)
	no response	42.11% (32/76)

SL: Second Life.

**Table 4:** Opinion questionnaire after clinical sessions: Second Life as an teaching environment and comparison with other face-to-face and distance methods

**Question**	**Answer**	**% (n/N)**
Does it matter to you to be represented by an avatar?	“it doesn’t matter”, “we aren’t aware of it”	32.89% (25/76)
	“no”, “not in the least”, “it’s fun”	35.53% (27/76)
	no response	31.58% (24/76)
Did you think it was positive being able to see the speaker, setting and presentations?	“yes”, “positive”	26.32% (20/76)
	“we only saw the screen”	47.36% (36/76)
	“we didn’t care”, “it didn’t matter”	26.32% (20/76)
In general, did you like this tool?	“yes”, “it let us connect with other centres in the zone”	100% (76/76)
Do you consider it better than other distance learning methods?	“yes”, “much better”, “by far”	65.79% (50/76)
	no response	32.21% (26/76)
Do you consider it better than other face-to-face learning methods?	“yes, in some aspects”, “it's more comfortable”	38.16% (29/76)
	“it depends”	2.63% (2/76)
	“no”	57.89% (44/76)
	no response	1.32% (1/76)

SL: Second Life.

**Table 5:** Opinion questionnaire after clinical sessions: Strengths and weaknesses of Second Life

**Question**	**Answer**	**% (n/N)**
What are the strengths of this tool?	it eliminates the need to travel	73.68% (56/76)
	more effective use of resources	68.42% (52/76)
	Improved access	47.37% (36/76)
What are the strengths of this tool?	technical (a great deal of updates, unstable connections, computer failure)	90.76% (69/76)
	impersonal and little interaction	9.21% (7/76)

SL: Second Life.

There was an uneven response. Of the 20 questions, 10 received a 100% response. The response rate for the other 10 questions ranged between 1.32% (1/76) and 42.11% (32/76).

## Discussion

A general lack of familiarity with SL was detected at the commencement of this project, despite being a tool used by more than 300 colleges and universities (Oxford [14], Carlos III [15]) and institutions (Instituto Cervantes [16], CDC [17]) for hosting regular events, seminars and workshops [18].

With respect to the opinion expressed by healthcare professionals about the virtual environment, we coincide with that of Wiecha et al [1], in which all the healthcare professionals surveyed consider SL to be superior to other distance learning methods (almost two thirds of the subjects in our study). However, we were surprised to find that in their study the majority also considered SL to be equal or superior to face-to-face methods, an opinion given by less than half of the subjects in our study. Although Wiecha’s study did not refer to acceptance, this is a possible explanation for the difference. It took a great deal of effort to have SL accepted as a useful teaching tool in our setting, despite the fact that the individuals involved were healthcare professionals under the age of 50 who had all had previous exposure to technology. This contrasts with the situation experienced by Holzinger [19], where lack of acceptance was related to lack of previous exposure to technology.

Nonetheless, our results do coincide with those presented by Wiecha in that the subjects liked it as a tool and were willing to repeat the experience.

One advantage observed was that when one health centre was connected through a single avatar (the one projected on the screen of the health centre library/seminar room), it did not cause the problems that occur when too many people are online at the same moment in real time with a single Internet access. However, some of the participants considered this to be a negative point, as it hindered the interaction that might have existed had they been given the use of individual computers.

The clinical sessions always began with theory, wherever this was applicable to the topic, followed by the presentation. Previous studies on simulations, such as that by Holzinger [20] showed that the successful use of simulations requires students to have a certain level of prior theoretical knowledge.

The advantages offered by SL according to all the participants was the opportunity it afforded them to interact with other health centres and with teachers, and that of facilitating the exchange of medical information between healthcare professionals. On the contrary, there was considered to be little interaction with other healthcare professionals, despite the possibility for this. This could be explained by the way the sessions were set up; in order to provide access to all health centre personnel, individual access could not be provided. One person entered SL from a single computer and image and sound were projected for the other participants. The results were not compared to those of other non-interactive training methods, such as platforms for simulating patient cases [21].

Most of the limitations of SL as a platform were found to be of a technical nature. In fact, technical problems were considered to be the main weakness of SL. One significant problem was that of continuous updates (as many as three in one month), with the consequence that SL could not be accessed if the updates had not been downloaded and installed. This was in addition to the problems caused by firewalls and anti-virus protection on health centre computers that prevent the downloading and installation of computer programs of unknown origin. When presented with an update, those health centres affected had to contact the IT service to update the version of SL in order to have access to the corresponding clinical session. Other technical problems related to sound and image that had caused major disruption during the trial sessions were completely resolved or minimized by the time the official programme began as a result of the experience gained by the participants.

Incidents were experienced involving the appearance of intruders in the virtual open-air auditorium during the course of some sessions. These included a professor of architecture from Madrid who was interested in incorporating this resource in his lectures, and general practitioners from other regions of Spain who were interested in using SL in teaching or incorporating SL into the accident and emergency services of a number of different hospitals. All of these intruders shared a teaching role and the space where the sessions were held.

A limitation of this study could be the way the questions in the survey were answered. As responses were given orally in SL, we feel this may have led to a number of responses differing from what they might have been if they had been obtained individually, anonymously or in writing. 10 of the questions were answered by only 42% of those present, fewer than half of the subjects surveyed, which may limit their inference.

Training in and use of SL currently covers 75% of urban health centres, 33% of rural health centres and the primary care pharmacy service in the zone. All junior doctors serving in the zone are currently receiving training in the use of SL. This will allow them to attend clinical sessions without the need to travel during their rotations with tutors in health centres and hospitals. Medical students on placements at health centres also participate in the clinical sessions in SL.

A third edition of the project will provide training for the remaining health centres in order to achieve 100% coverage, and for health professionals at the zone’s referral hospital.

## Conclusions

SL is a tool that enables participation and the effective use of educational resources. It eliminates the need for travel and improves access to educational activities for primary healthcare professionals in different geographical locations.

## Competing interests

All the authors declare that they have no competing interests.

## Authors’ contributions

EMP was jointly responsible for the Second Life training workshops, the research project and statistical study, and for writing the manuscript. CBM was responsible for the Second Life training workshops, the research project and statistical study, and for drafting the manuscript. JPA participated in preparing the clinical sessions for CPD accreditation and in drafting the manuscript. ALL participated in the research project and in drafting the manuscript. IGG was jointly responsible for the Second Life training workshops, the research project, the statistical study and for writing the manuscript. SG participated in the clinical sessions and in drafting the manuscript. ABE participated in the clinical sessions and in drafting the manuscript. SC participated in the clinical sessions and in drafting the manuscript. AMM participated in the clinical sessions and in drafting the manuscript. AMM participated in the clinical sessions and in drafting the manuscript. CL participated in the clinical sessions and in drafting the manuscript. RMB participated in the clinical sessions, the research project and he drafting of the manuscript. All authors read and approved the final manuscript.

## Pre-publication history

The pre-publication history for this paper can be accessed here:

http://www.biomedcentral.com/1472-6920/12/30/prepub
